# The Role of Olive Oil in Cardiometabolic Risk

**DOI:** 10.3390/metabo15030190

**Published:** 2025-03-11

**Authors:** Andrea Salvo, Antonino Tuttolomondo

**Affiliations:** 1 Internal Medicine and Stroke Care Ward, Policlinico, P. Giaccone, Piazza delle Cliniche n.2, 90127 Palermo, Italy; andrea.salvo996@gmail.com; 2 ProMISE Department, University of Palermo, 90127 Palermo, Italy

**Keywords:** olive oil, cardiovascular disease, Mediterranean diet

## Abstract

Olive oil, the primary fat source in the Mediterranean diet (MedDiet), is rich in monounsaturated fatty acids (MUFA), especially oleic acid, which constitutes 70–80% of its composition. Extra-virgin olive oil (EVOO), produced by mechanically pressing olives, is the highest quality olive oil, with an intense flavor and acidity <1%. In contrast, refined olive oil (ROO), a blend of virgin and refined oils, contains fewer antioxidants and anti-inflammatory compounds. EVOO’s health benefits stem largely from its MUFA content, which is linked to reduced risks of cardiovascular disease (CVD), neurodegenerative conditions, and certain cancers. Additionally, EVOO contains minor, but bioactive, components such as polyphenols, tocopherols, and phytosterols, contributing to its oxidative stability, sensory qualities, and health-promoting properties. These include polyphenols, like oleuropein, hydroxytyrosol, and tyrosol, which exhibit anti-inflammatory, cardioprotective, neuroprotective, and anticancer effects. Epidemiological studies suggest an inverse relationship between olive oil intake and CVD, with EVOO-enriched MedDiet interventions showing improved lipid profiles, reduced blood pressure, and lower cardiovascular event risk. The PREDIMED study highlights the significant role of EVOO in reducing cardiometabolic risk. This review explores the impact of EVOO’s chemical components within the MedDiet framework on metabolic variables influencing cardiometabolic health.

## 1. Introduction

The Mediterranean diet, distinguished by its prominent use of olive oil (OO), has been linked to numerous health benefits, largely attributed to its bioactive compounds. Derived from *Olea europaea* L., a fruit tree native to Asia Minor and Syria, olive cultivation has expanded widely across the Mediterranean region. Today, Spain, Italy, and Greece are the world’s top producers of olives and olive oil. Extra-virgin olive oil (EVOO), extracted solely through mechanical means, is celebrated for its superior nutritional composition and its protective effects against cardiovascular diseases (CVDs), making it a cornerstone of heart-healthy dietary practices [1]. Unlike other dietary oils, which often require chemical processing and extraction, extra-virgin olive oil (EVOO) is produced through mechanical methods, preserving its valuable components. Its health-promoting properties are largely attributed to its high fatty acid content, which constitutes 98–99% of its total weight, predominantly monounsaturated fatty acids such as oleic acid. Additionally, EVOO contains bioactive compounds in smaller amounts (1–2%), including phenolics, phytosterols, tocopherols, and squalene. Unlike seed oils, EVOO boasts a high proportion of fatty acids with an optimal balance of unsaturated fats, further stabilized by minor polar compounds with strong antioxidant properties, enhancing its nutritional and therapeutic value [2].

### 1.1. Olive Oil Compounds

Olive oil is a rich source of monounsaturated fatty acids (MUFAs), predominantly oleic acid, and contains a variety of unsaponifiable and hydrophilic compounds that contribute to its health benefits. The unsaponifiable fraction includes phytosterols, triterpenes, squalene, and pigments, while the hydrophilic fraction features polyphenols and tocopherols. Among the minor polar compounds, several subclasses are noteworthy. Secoiridoids represent a prominent group, including the dialdehydic form of decarboxymethyl elenolic acid linked to ortho-diphenolic and/or phenolic alcohols, such as oleuropein aglycone (OLE), oleacein, deacetoxyoleuropein, and oleocanthal (OLC), as well as traces of ligstroside aglycone. Phenolic alcohols, such as hydroxytyrosol (HT) and tyrosol (TYR), are also present alongside their secoiridoid precursors. Additionally, EVOO contains traces of phenolic acids, including gallic acid, protocatechic acid, p-hydroxybenzoic acid, vanillic acid, caffeic acid, syringic acid, p- and o-coumaric acid, ferulic acid, and cinnamic acid. Flavonoids are present in small quantities, with luteolin and apigenin being the most abundant flavones. Lastly, the lignan class includes acetoxypinoresinol and pinoresinol as the most representative compounds in EVOO. These bioactive components collectively contribute to the oil’s antioxidant, anti-inflammatory, and cardioprotective properties [3]. Monounsaturated fatty acids (MUFAs) and polyphenols, particularly oleuropein, hydroxytyrosol, and tyrosol, are key bioactive compounds that underpin olive oil’s protective effects against the development of various diseases [4,5]. Epidemiological studies consistently link higher olive oil consumption with a decreased risk of numerous chronic conditions [6]. Findings from multiple landmark studies highlight the broad health benefits of olive oil, with its positive impact observed in cardiovascular disease prevention, cancer risk reduction, type 2 diabetes management, body composition improvement, blood pressure regulation, inflammation control, enhancement of endothelial function, and the maintenance of hemostatic balance [7]. This effect are summarized in Table 1.

### 1.2. Main Effect of Olive Oil Compounds and Principle Supporting Study

These effects underscore the central role of olive oil in promoting long-term health and preventing chronic diseases. In 2011, the European Food Safety Authority (EFSA) approved specific health claims recognizing the beneficial properties of bioactive compounds found in foods, with a particular focus on the phenolic compounds in extra-virgin olive oil (EVOO), such as hydroxytyrosol (HT) and oleuropein (OLE). These endorsements underscore their substantial role in supporting human health. The cardioprotective effects of the Mediterranean diet (MedDiet), primarily attributed to the health benefits of EVOO, were first evidenced in the landmark Seven Countries Study of Cardiovascular Disease (SCSCD). This study provided foundational insights into the diet’s role in reducing cardiovascular risk [8].

Extra-virgin olive oil (EVOO) and refined olive oil differ significantly in both chemical composition and health benefits. Extra-virgin olive oil (EVOO) is obtained through cold mechanical extraction, and retains a high concentration of phenolic compounds and natural antioxidants. Additionally, EVOO is rich in monounsaturated fatty acids (MUFAs), primarily oleic acid, which contribute to its cardioprotective effects [9]. Refined olive oil is subjected to refining processes that involve high temperatures and chemical agents, and undergoes a significant reduction in phenolic compounds and antioxidants. Consequently, despite maintaining a similar lipid profile, refined olive oil has a lower concentration of bioactive components with health-promoting properties [10]. Several studies have demonstrated that EVOO consumption is associated with a reduction in cardiovascular risk factors. For instance, research has shown that EVOO intake lowers blood pressure and body fat mass, suggesting its role in the prevention of obesity and cardiovascular diseases [11]. Moreover, EVOO has been found to exert beneficial effects on gut microbiota, positively modulating bacterial composition and contributing to metabolic health [1]. Due to the depletion of phenolic compounds and antioxidants during processing, refined olive oil offers fewer health benefits than EVOO.

EVOO is distinguished by its high polyphenol content, which contributes substantially to its antioxidant and anti-inflammatory properties. For example, Covas et al. demonstrated that the polyphenolic fraction of EVOO improves endothelial function and reduces oxidative stress, likely through modulation of lipid oxidation and inflammatory pathways [12]. In contrast, refined olive oil and other vegetable oils such as sunflower oil possess markedly lower concentrations of these bioactive compounds, potentially limiting their cardioprotective effects [13]. Oils such as fish oil, which are rich in omega-3 polyunsaturated fatty acids, primarily exert their beneficial effects by modulating inflammatory cascades and eicosanoid synthesis [14]. This represents a distinct mechanistic pathway compared to the antioxidant and anti-inflammatory actions mediated by EVOO polyphenols. Moreover, while canola oil and other seed oils are high in monounsaturated fatty acids, they lack the diverse polyphenolic profile characteristic of EVOO.

The PREDIMED study, that is an interventional study, examined the protective effects of the Mediterranean diet (MD), supplemented with either extra-virgin olive oil (EVOO) or nuts, on major cardiovascular (CV) events in a cohort of 7477 individuals at high risk of cardiovascular diseases (CVDs). The findings revealed that participants adhering to the MD enriched with EVOO or nuts experienced a significantly lower incidence of major CV events, including stroke, myocardial infarction, and CV-related mortality, compared to those following a reduced-fat diet. These results underscore the efficacy of the MD in the primary prevention of cardiovascular disease [15].

**Figure 1:**
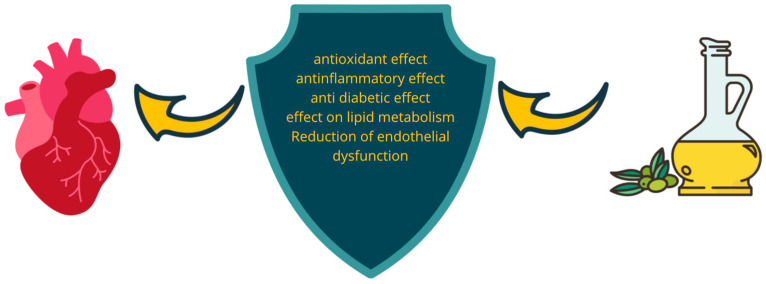
Effects of olive oil on cardiovascular health.

The EPICOR study, a long-term investigation into antithrombotic management patterns in acute coronary syndrome patients, which is an observational study, enrolled 29,689 Italian women from northern, central, and southern regions to assess the associations between the consumption of extra-virgin olive oil (EVOO), vegetables, and fruit, and the incidence of coronary heart disease (CHD). Over a mean follow-up period of 7.85 years, the study found that women with the highest quartile intake of vegetables and olive oil exhibited a significantly reduced risk of developing CHD. These findings reinforce the protective role of vegetables and olive oil in both the primary and secondary prevention of cardiovascular diseases [16]. Figure 1 summarizes the main mechanisms underlying the cardiovascular protection provided by olive oil.

## 2. Hypolipidemic Effect

The hypolipidemic properties of olive oil, especially extra-virgin olive oil (EVOO), have attracted considerable interest within the scientific community for their ability to favorably influence lipid profiles and lower cardiovascular risk [17]. The lipid-lowering effects of olive oil, particularly extra-virgin olive oil (EVOO), are primarily attributed to its distinctive composition, which includes monounsaturated fatty acids (MUFAs) and a variety of bioactive compounds, such as polyphenols. Compounds like hydroxytyrosol (HT) and oleuropein (OLE) have been extensively studied for their roles in modulating lipid metabolism, reducing oxidation of low-density lipoprotein (LDL) cholesterol, and enhancing the functionality of high-density lipoprotein (HDL) cholesterol [1]. These properties highlight olive oil’s therapeutic potential as a fundamental component of a heart-healthy diet. EVOO’s hypolipidemic effects and its cardioprotective role result from a combination of distinct biological mechanisms. A key action is its ability to protect HDL cholesterol from oxidation and to enhance cholesterol efflux—the initial step of reverse cholesterol transport.

Farràs et al. were the first to provide evidence that consuming a practical dose of virgin olive oil (VOO) at 366 mg/kg significantly improved cholesterol efflux in humans. Additionally, research from this group demonstrated that functional virgin olive oil with thyme (FVOOT, 500 ppm) increased cholesterol efflux compared to an olive oil polyphenol concentrate (OOPC)-enriched VOO (500 ppm) in hypercholesterolemic patients. However, this enhancement was not observed when compared to baseline levels or a natural VOO, suggesting a nuanced effect dependent on specific formulations and baseline conditions [18]. Additionally, in this regard, few studies have analyzed the direct effect of VOO on the cholesterol efflux-related gene expression profile. Hydroxytyrosol, the main PC from OO, has been demonstrated to enhance the peroxisome proliferator-activated receptors (PPARα and PPARγ) of gene expression in 3T3-L1 adipocytes [19]. An increase in cholesterol efflux-related gene expression in peripheral blood mononuclear cells was observed following an intervention with functional virgin olive oil with thyme (FVOOT, 500 ppm) in hypercholesterolemic individuals.

Specifically, FVOOT upregulated genes such as CYP27A1, CAV1, LXRβ, RXRα, and PPARβ/δ. Additionally, gene expression of cholesterol efflux transporter genes, including *ABCA1*, scavenger receptor B1 (*SCARB1*), and several transcription factors associated with peroxisome proliferator-activated receptors (*PPARα*, *PPARγ*, *PPARβ/δ*, and *MED1*), increased following an acute intervention with an olive oil polyphenol concentrate (OOPC)-enriched VOO (961 mg/kg) in pre- and hypertensive patients compared to a control VOO (289 mg/kg). Collectively, these findings suggest that both VOO and polyphenol-enriched VOOs can upregulate a range of genes involved in regulating macrophage cholesterol efflux, thereby potentially contributing to improved lipid metabolism and cardiovascular health [20]. High-density lipoproteins (HDLs) can serve as transporters for various derivatives of olive oil polyphenol concentrates (OOPCs) to endothelial cells. Once delivered, these compounds may help prevent oxidative damage in the cell mitochondria and preserve nitric oxide (NO) production, as demonstrated in in vitro studies. This mechanism highlights the potential of HDLs enriched with OOPC derivatives in protecting endothelial function and supporting vascular health [21,22].

Another mechanism by which olive oil can reduce cholesterol levels is by acting on LDL oxidation. The atherogenicity of low-density lipoprotein (LDL) is primarily determined by factors such as LDL particle size, the extent of oxidative modifications, resistance to oxidation, and cytotoxicity. The LDL particle number (LDL-P) to high-density lipoprotein particle number (HDL-P) ratio has the strongest independent association with cardiovascular disease (CVD). Oxidized LDLs (OxLDLs), when bound to the LOX-1 receptor (lectin-like oxidized LDL receptor-1), stimulate endothelial cells to express and secrete pro-atherogenic enzymes. This interaction leads to the production of superoxide and a reduction in local nitric oxide (NO) concentrations [23]. LOX-1, a specific endothelial scavenger receptor, triggers a rapid increase in reactive oxygen species (ROS) levels through membrane-bound NADPH oxidase (NOX), playing a crucial role in the initial stages of atherosclerotic plaque formation [23]. A recent randomized, double-blind, controlled crossover trial demonstrated that the administration of hydroxytyrosol (HT, 3.3 mg) in combination with punicalagin (65 mg), a phenolic compound from pomegranate, significantly reduced plasma triglyceride and LDL cholesterol (LDL-C) levels. This study involved eighty-four hypercholesterolemic participants (both male and female), who took three capsules daily (HT + punicalagin + 331.7 mg maltodextrin) for a duration of eight weeks. These findings suggest a potential synergistic effect of HT and punicalagin in improving lipid profiles [24]. One proposed mechanism for the lipid-lowering effects of hydroxytyrosol (HT) and punicalagin involves the modulation of cholesterol metabolism. This occurs through the reduction in MAPK p38 phosphorylation, which subsequently activates AMP-activated protein kinase (AMPK) and inhibits nuclear factor-kappa B (NF-κB). These signaling changes lead to the upregulation of the LDL receptor (LDLR) and the inhibition of the sterol regulatory element-binding protein 2/proprotein convertase subtilisin/kexin type 9 (SREBP2/PCSK9) pathway. This cascade of events helps to enhance LDL cholesterol clearance and improve lipid profiles [25]. A traditional Mediterranean diet (MD) enriched with extra-virgin olive oil (EVOO) was tested in humans at high cardiovascular risk. The intervention led to a reduction in oxidized LDL (oxLDL), improved LDL resistance to oxidation, a decrease in the degree of LDL oxidative modifications, an increase in LDL particle size, and a reduction in LDL particle cytotoxicity. However, in the same study, a traditional MD enriched with nuts did not produce any significant changes in these LDL characteristics. This suggests that EVOO may have a more pronounced effect on improving LDL profile compared to nuts in the context of cardiovascular risk management [26,27]. In the Eurolive study [28], which is a long term interventional study, a natural extra-virgin olive oil (EVOO) rich in polyphenols (366 mg/Kg) led to a decrease in plasma apoB-100 concentrations and a reduction in the number of total and small LDL particles. In contrast, virgin olive oil (VOO), with a low phenolic content, resulted in an increase in these parameters in healthy volunteers. Additionally, the low-phenolic VOO also enhanced LDL resistance to oxidation. These findings highlight the role of polyphenol content in EVOO in modulating lipid profiles and oxidative stress [29].

## 3. Hypoglycemic Effect

Diabetes mellitus (DM) is considered a significant public health issue and one of the most prevalent metabolic disorders, affecting millions of individuals worldwide. A small-scale study involving overweight type 2 diabetes (T2D) patients demonstrated that intake of extra-virgin olive oil (EVOO) (equivalent to 577 mg/kg, predominantly as hydroxytyrosol or HT) resulted in reduced fasting plasma glucose concentrations, lower glycated hemoglobin A1c (HbA1c) levels, decreased body weight, and reduced inflammatory adipokines [30,31]. A nested substudy of the PREDIMED trial showed that, after a median follow-up of 4 years, there was a 51% reduction in type 2 diabetes (T2D) rates in individuals who followed a Mediterranean diet (MedDiet) enriched with extra-virgin olive oil (EVOO) compared to those on a low-fat diet. This suggests the potential role of EVOO in the prevention of diabetes [32]. Santangelo et al. were the first to observe that daily consumption of polyphenol-rich extra-virgin olive oil (EVOO) is associated with a reduction in fasting plasma glucose (FPG) levels and glycated hemoglobin (HbA1c). This effect is likely due to a decrease in visfatin levels, an adipose tissue-derived hormone that acts as a pro-inflammatory cytokine and plays a key role in impaired glucose metabolism [30].

Phenolics may exert potential anti-diabetic effects due to their strong free radical scavenging and antioxidative properties. Animal models and in vitro evidence suggest their interaction with intracellular signaling pathways, such as the nuclear transcription factor (erythroid-derived 2)-like 2 (Nrf2), which regulates the expression of antioxidant proteins that protect against oxidative damage. In vitro studies indicate that hydroxytyrosol (HT) and oleuropein may play a protective role against oxidative stress by activating the Nrf2/ARE pathway in a dose-dependent manner. HT, in particular, demonstrates potent radical scavenging activity and can upregulate protective enzymes, including thioredoxin reductase [33]. Oleuropein, one of the most abundant polyphenols in extra-virgin olive oil (EVOO), promotes beta cell insulin secretion and suppresses cytotoxicity caused by amylin amyloids. The aggregation of these amyloids is associated with β-cell dysfunction, and oleuropein appears to mitigate this harmful process, potentially contributing to improved insulin production and overall pancreatic health [34,35]. Another well-known phenolic compound, tyrosol, has been shown to prevent endoplasmic reticulum stress-induced apoptosis in beta cells. This protective effect is mediated by its interference with the Jun N-terminal kinase (JNK) signaling pathway, which plays a crucial role in the regulation of cellular stress responses and apoptosis [36]. In addition to their effects on pancreatic beta cells, the phenolic compounds in extra-virgin olive oil (EVOO) also contribute to the inhibition of α-amylase and α-glucosidase. This inhibition helps to control postprandial hyperglycemia by delaying the absorption of carbohydrates, thus reducing the spike in blood glucose levels after meals [37,38]. Carnevale et al. demonstrated that oleuropein improved postprandial glycemic control by interfering with the activity of soluble NADPH oxidase-derived peptide (sNox2-dp). Postprandial activation of Nox2 leads to elevated levels of reactive oxygen species (ROS), which play a key role in the incretin phenomenon. Consequently, oleuropein, and by extension extra-virgin olive oil (EVOO), may function as dipeptidyl-peptidase 4 (DPP-4) inhibitors. This occurs through the inhibition of DPP-4 production and the enhancement of glucagon-like peptide-1 (GLP-1) activity, which helps regulate blood glucose levels [39]. Bartimoccia et al. demonstrated that the addition of extra-virgin olive oil (EVOO) to a Mediterranean diet (MedDiet) or chocolate altered gut permeability, thereby reducing metabolic endotoxemia. This effect was achieved by lowering circulating levels of lipopolysaccharides (LPS) and zonulin, a protein that increases the permeability of tight intestinal junctions. Notably, the levels of zonulin were inversely associated with glucagon-like peptide-1 (GLP-1) levels, suggesting that EVOO may help modulate gut health and improve metabolic outcomes through this mechanism [1,2,3,4,5,6,7,8,9,10,11,12,13,14,15,16,17,18,19,20,21,22,23,24,25,26,27,28,29,30,31,32,33,34,35,36,37,38,39,40].

A recent meta-analysis on olive oil (OO) consumption in type 2 diabetes (T2D) patients found that it was associated with a lower production of advanced glycosylated end-products (AGEs). These compounds are typically formed when proteins or lipids become glycated as a result of high blood sugar levels, and their accumulation is linked to the development and progression of diabetes-related complications. This evidence suggests that olive oil, particularly when included in a balanced diet, may help mitigate some of the harmful effects associated with chronic hyperglycemia in T2D [41]. Hydroxytyrosol (HT) supplementation (10 mg/kg/day for 5 weeks) was shown to enhance glucose tolerance and improve insulin sensitivity in rat models. This led to a decrease in the homeostatic model assessment of insulin resistance (HOMA-IR), a commonly used measure of insulin resistance. These findings suggest that HT, a major phenolic compound in olive oil, may have potential therapeutic effects in improving insulin function and glucose metabolism [42].

Further potential anti-diabetic mechanisms have been demonstrated in experimental in vitro studies for various polyphenolic compounds, including flavonoids, chlorogenic acid, ferulic acid, caffeic acid, and tannic acid. These compounds have been shown to inhibit key enzymes involved in carbohydrate digestion, such as α-amylase and α-glucosidase, as well as the sodium-dependent SGLT1-mediated glucose transporter. By inhibiting these enzymes and transporters, these polyphenols may influence glucose metabolism and help regulate blood sugar levels by delaying carbohydrate digestion and absorption [43,44]. Hydroxytyrosol (HT) has a high degree of bioavailability, with absorption rates ranging from 40 to 95% in humans following the ingestion of EVOO. Once consumed, oleuropein glycoside, oleuropein, and ligstroside aglycones are converted into HT or tyrosol, which are then excreted in the urine. These compounds are sometimes conjugated with glucuronic acid and excreted as glucuronides. The ingestion of EVOO, in its oil form, might also help mitigate the breakdown of these bioactive compounds in the gastrointestinal tract. The mechanisms behind the absorption and effects of key bioactive compounds from olive oil may explain their beneficial impact in preventing and managing type 2 diabetes (T2D) and other cardiometabolic diseases. It is likely that biophenols, such as HT, influence glucose metabolism through multiple pathways, including the inhibition of carbohydrate digestion and glucose absorption in the intestine, activation of insulin receptors, enhancement of glucose uptake in tissues, upregulation of GLP-1 [45], reduction in DPP-4 levels [46], antioxidative properties, and immunomodulatory effects [47].

The beneficial effects of olive oil and its bioactive compounds, particularly oleuropein, include several mechanisms that contribute to the prevention and management of metabolic disorders such as type 2 diabetes. Oleuropein has been shown to reduce amyloid aggregation and prevent inflammation and cytokine-induced oxidative damage in pancreatic β-cells, thus enhancing their capacity to secrete insulin. Olive leaf extracts also offer benefits by lowering glucose and cholesterol levels. These extracts modify gene expression related to lipogenesis, thermogenesis, and insulin resistance. Furthermore, they reduce the digestion and intestinal absorption of dietary carbohydrates on both the mucosal and serosal sides of the intestine. In addition, olive oil consumption has been linked to reductions in HbA1c and fasting plasma insulin levels. It also acutely enhances insulin sensitivity and related gene expression, contributing to improved glucose metabolism [48]. The synergistic effects between healthy food components, such as olive oil (OO), and other lifestyle factors, particularly physical activity, play a crucial role in the prevention and management of type 2 diabetes (T2D). The combination of a balanced diet rich in bioactive compounds, including those found in olive oil, and regular physical exercise, can enhance insulin sensitivity, improve glucose metabolism, and reduce inflammation. This integrated approach supports overall metabolic health, helping to manage blood sugar levels and reduce the risk of complications associated with T2D [49]. Enhancing the anabolic effects of strength training through the novel effects of olive oil (OO) compounds is an intriguing area in the prevention of Type 2 diabetes (T2D), particularly considering the importance of strength training for T2D patients or those at high risk. Recent animal studies have highlighted the potential anabolic-enhancing effects of OO biophenols, particularly on androgen function. For instance, oleuropein supplementation was shown to increase testicular testosterone concentrations, reduce plasma corticosterone levels, and enhance plasma luteinizing hormone (LH) in rat models. These findings suggest that OO biophenols may play a role in promoting muscle mass and strength, which could be beneficial in managing T2D, as increased muscle mass improves insulin sensitivity and overall metabolic health [50]. These anabolic effects were observed following the addition of 0.1 g per 100 g oleuropein to a high-protein (40%) diet (40, 25, and 10 g per 100 g casein) for 28 days [51,52]. For example, testosterone deficiency promotes insulin resistance and increases the risk of T2D [53,54].

Testosterone plays a critical role in the regulation of body composition in males and exhibits potential anti-obesity effects, mediated by the androgen receptor (AR) [55]. Emerging research from knockout mice suggests that androgen receptor (AR) signaling in adipocytes plays a protective role in regulating insulin action and glucose homeostasis, independent of adiposity. This finding indicates that AR signaling may influence metabolic processes in adipocytes in a way that improves insulin sensitivity and helps maintain glucose balance, regardless of the amount of body fat present. These results highlight the potential importance of AR signaling in metabolic health, particularly in the context of conditions like type 2 diabetes, where insulin resistance and glucose dysregulation are central concerns [56].

## 4. Anti-Inflammatory Effect

Diet and dietary supplements can, indeed, play a role in modulating the secretion of inflammatory cytokines. Studies indicate that compounds like oleuropein (OLE) and hydroxytyrosol (HT) found in olive oil have significant effects on both pro-inflammatory and anti-inflammatory pathways. These effects occur at both the local level (such as within tissues) and the systemic level (impacting broader bodily functions). OLE and HT have been shown to downregulate the secretion of pro-inflammatory cytokines while promoting the production of anti-inflammatory mediators, which may contribute to their overall cardiovascular and metabolic health benefits [57]. Experimental evidence is analyzed in the light of cellular pathways ruling low-grade chronic inflammation.

Actually, the acronym IL-1 is used to indicate two cytokines encoded by separated genes and secreted by both innate and adaptive immune cells, IL-1α and IL-1β [58], in older subjects, increased IL-1 levels are associated with an augmented risk of mortality and morbidity, atherosclerosis, and type 2 diabetes [59]. As demonstrated in vitro, OLE acts on both IL-1β release and IL-1β-mediated inflammatory action. An OLE-rich extract (OLE concentration = 379 mg/g) reduced IL-1β expression in LPS-stimulated RAW264.7 cells in a time-dependent manner [60]. Pre-treatment with 300 μM oleuropein (OLE) in LPS-stimulated RAW264.7 macrophages led to a significant reduction in IL-1β expression at both the mRNA and protein levels. This effect was attributed to the inhibition of IκB-α phosphorylation and the subsequent prevention of NF-κB nuclear translocation [61]. Similarly, OLE counteracted IL-1β-induced inflammation via the suppression of NF-κB and MAPK signaling [62]. In vivo, OLE reduced serum IL-1β in LPS-induced sepsis, decreasing NF-κB mRNA levels [63]. In a myocardial ischemia/reperfusion model, the reduction in serum IL-1β was associated with the decrease in protein levels of phospho-IκBα (p-IκBα, an inhibitor of NF-κB), as well as the kinases phospho-MEK (p-MEK) and phospho-ERK (p-ERK). Additionally, this reduction correlated with alterations in the transcription factor p53, which regulates cell survival and apoptosis [64]. An analogous reduction in IL-1β levels elicited by OLE was obtained in serum in a heart failure model [65,66]. Experiments conducted in a high-fat diet model demonstrated that the reduction in IL-1β mediated by olive leaf extracts (containing 10% OLE) is primarily regulated at the transcriptional level, particularly in the liver and adipose tissues [67].

Further studies on renal tissue identified a mechanism mediated by HT that may prevent the degradation of IκBα, inhibit the phosphorylation of MAPK, and block the nuclear translocation of p65, a subunit of NF-κB. Additionally, a study in apoE−/− mice confirmed that HT treatment resulted in a reduction in phosphorylated forms of p38 MAPK and NF-κB in the liver, alongside a decrease in serum IL-1β levels [68].

IL-6 is a pleiotropic interleukin that functions as a pro-inflammatory mediator when produced by senescent and immune cells through NF-κB- and TNF-α-dependent pathways. The levels of IL-6 are closely linked to aging, morbidity, mortality, and elevated serum concentrations of C-reactive protein (CRP) [69]. Prospective studies have demonstrated that IL-6 levels are associated with an increased risk of coronary heart disease [70]. In vitro, long-term treatment (4–6 weeks) of pre-senescent human fetal lung fibroblasts and human neonatal lung fibroblasts with 1 μM HT and 10 μM OLE resulted in a reduction in IL-6 release compared to senescent untreated cells. Statistically significant effects were observed with HT in both cellular models [71]; pre-treatment with 50 μM and 100 μM HT effectively downregulated IL-6 expression and cytokine release in LPS-stimulated RAW264.7 macrophages [72]. The molecular mechanisms induced by OLE and HT appear to be closely linked to the pro-inflammatory stimuli that trigger IL-6 secretion. Both HT and OLE were found to reduce TNF-α-stimulated IL-6 release in murine osteoblast-like cells, likely through the suppression of TNF-α-induced phosphorylation of p44/p42 MAPK and AKT, a serine/threonine kinase involved in ROS homeostasis. Additionally, HT was shown to mediate the downregulation of IL-6 transcription [73,74], additionally, OLE interferes with LPS-induced Toll-like receptor 4 (TLR4) dimerization, thereby alleviating inflammation by disrupting the TLR4-MyD88-NF-κB/MAPK signaling axis [75] in an in vivo model of myocardial ischemia/reperfusion, and in experimental autoimmune myocarditis [64,65,66,67,68,69,70,71,72,73,74,75,76].

TNF-α is a transmembrane protein expressed by a variety of cell types, including macrophages, dendritic cells, and senescent cells, among others. It is cleaved by tumor necrosis factor converting enzyme (TACE) and released in response to inflammatory stimuli. Both the transmembrane and soluble forms of TNF-α bind to TNF-α receptors TNFR1 (CD120a) and TNFR2 (CD120b) with varying affinities. This binding promotes a pro-inflammatory environment by activating NF-κB and MAPK pathways, thereby regulating the synthesis of adhesion molecules and soluble mediators. Additionally, TNF-α plays a crucial role in the regulation of cell proliferation and the processes of apoptosis and necrosis [77,78]. In vitro, murine J774 macrophages were pre-treated with OLEsus, NLC-OLE, and blank (unloaded) nanoparticles (NLC-BLANK) before stimulation with LPS. While OLEsus did not reduce TNF-α levels, both nanoparticle formulations (NLC-OLE and NLC-BLANK) decreased TNF-α levels, regardless of whether OLE was present. This finding suggests that the reduction in TNF-α observed in this experimental context is not attributable to the biological activity of OLE [79]. In vivo data for HT are more consistently reproduced. Notably, 200 mg/L HT reduced circulating TNF-α levels in cyclophosphamide-induced immunosuppressed broilers [80] and in a mouse model of LPS-induced systemic inflammation [81], and HT administration diminished serum TNF-α in apoE−/− mice together with a reduction in p38 MAPK and NF-κB phosphorylation in liver [68]. HT-rich extracts reduced TNF-α secretion in both plasma and liver in an in vivo model of high-fat diet [82].

Selectins are key adhesion molecules in the inflammatory process, expressed on the surfaces of activated endothelial cells, platelets, and leukocytes in response to stimulation by TNF-α, IL-6, and other pro-inflammatory cytokines. Elevated levels of E-selectin have been found to correlate with ischemic events [83], independently of traditional risk markers, as demonstrated in the PRIME study, which is an observational study [84,85]. ICAM-1 and VCAM-1 facilitate the adhesion of leukocytes to the endothelium and are both upregulated by pro-inflammatory cytokines. However, VCAM-1 is typically expressed in more advanced stages of atherosclerosis. Oleanolic acid (OLC), a component of extra-virgin olive oil (EVOO), is responsible for the characteristic pungency of this food. This sensory perception is thought to be mediated by the presence of a specific OLC receptor located in the oropharyngeal region [86]. This receptor is believed to be the transient receptor potential channel, subfamily A, member 1 (TRPA1). It is hypothesized that the varying sensitivity to the pungent taste of OLC may be linked to inter-individual differences in the expression of the TRPA1 receptor in the oropharynx [87]. This sensation is similar to that experienced after taking ibuprofen, a non-steroidal anti-inflammatory drug (NSAID). Based on this observation, some researchers have hypothesized that OLC and ibuprofen may share similar biological activities, potentially influencing inflammatory pathways in a comparable manner [88]. Despite structural differences, both OLC and ibuprofen inhibit the same cyclooxygenase enzymes involved in the biosynthesis of prostaglandins. Specifically, both enantiomers of OLC inhibit cyclooxygenase (COX) 1 and COX 2, but show no effect on lipoxygenase in vitro. The inhibition of COX 1 and COX 2 by OLC is dose-dependent. For instance, an OLC concentration of 25 μM inhibits COX activity by 41% and 57%, whereas 25 μM of ibuprofen inhibits COX activity by only 13% to 18%, respectively [89].

PCSK9 plays a pivotal role in regulating circulating cholesterol levels by reducing the number of membrane-associated LDL receptors. Studies have demonstrated that PCSK9 also increases systemic inflammation [90] and has been linked to the onset of respiratory and cardiovascular failure. Beyond lipid metabolism, PCSK9 is involved in a wide array of physiopathological processes, including inflammatory and stress responses, glucose metabolism, and cell apoptosis. Regarding its role in the inflammatory process, PCSK9 is known to stimulate the release of a range of chemokines and cytokines, particularly from macrophages, leading to increased infiltration and activation of monocytes [91,92]. Ikonomidis et al. found a reduction in CRP and PCSK9 levels and reduced IL-6 levels after treatment with OOHT compared to baseline [93].

IL-4 plays a key role in suppressing inflammation by directly inhibiting the production of pro-inflammatory cytokines in macrophages. It also promotes the differentiation of T helper type 2 (Th2) cells, which are involved in allergic responses and the secretion of anti-inflammatory mediators. Simultaneously, IL-4 suppresses the differentiation of T helper type 1 (Th1) cells, which are associated with inflammation and the promotion of immune responses [94].

In vivo data showed that 200 mg/L HT upregulated IL-4 at duodenal level in both HT-only-treated and in cyclophosphamide-induced immunosuppressed broilers [80], and that 100 mg/kg HT increased IL-4 serum levels in a model of acute liver injury [72]. IL-10 is the most extensively studied anti-inflammatory and immunomodulatory cytokine, playing a crucial role in exerting broad immunosuppressive effects at multiple levels. It regulates both innate and adaptive immune responses, including those mediated by Th2 cells. IL-10 helps to limit excessive immune activation and inflammation, contributing to immune homeostasis and preventing tissue damage [95]. In vitro, 41 μM HT increased IL-10 transcription and cytokine secretion in human monocytes after stimulation with LPS [96]. In vivo, OLE has been shown to affect plasma IL-10 levels in a model of sepsis-induced myocardial injury. This effect appears to be mediated, at least in part, by the suppression of NF-κB phosphorylation, which plays a critical role in the regulation of inflammatory responses [97].

Transforming growth factor beta (TGF-β) is crucial for maintaining homeostasis during immune responses and facilitating tissue repair following inflammation resolution. However, due to its role in immune differentiation, TGF-β is also implicated in pathological conditions such as fibrosis and age-related diseases, including atherosclerosis, obesity, and frailty. Its dual role in both repair and pathology highlights its complex involvement in immune regulation and tissue remodeling [98]. OLE increased TGF-β release by human isolated T cells [99].

## 5. Antioxidant Effect

The production of reactive oxygen species (ROS), both radical (such as superoxide anions, O•^−2^) and non-radical (such as hydrogen peroxide, H_2_O_2_), is a hallmark of aging, as well as numerous chronic and acute diseases, including diabetes mellitus [97]. Elevated levels of inflammatory and oxidative markers have been shown to be predictive of future cardiovascular events, highlighting the role of oxidative stress in cardiovascular risk and disease progression [100]. Both HT and OLE directly scavenge free radicals [101], OLE and HT demonstrated a concentration-dependent ability to reduce ROS production in human granulocytes challenged with phorbol myristate acetate (PMA). HT exhibited a similar ability to decrease ROS production in monocytes under the same culture conditions [102]. Experiments on pheochromocytoma PC12 cells [103] and human PBMCs [104] demonstrated that doses of HT, at concentrations ranging from 25 to 100 μM, reduced hypoxia-induced intracellular ROS levels and protected cells from oxidative damage induced by 2,3,7,8-tetrachlorodibenzo-p-dioxin. This protective effect was mediated by the activation of antioxidant enzymes, including superoxide dismutase (SOD), catalase (CAT), and glutathione peroxidase (GSH-Px), through the PI3K/Akt/mTOR-HIF-1α signaling pathway. This mechanism involved the upregulation of PI3K, phospho-Akt (p-Akt), and phospho-mTOR (p-mTOR) protein levels. Additionally, HT protected Jurkat cells from H_2_O_2_-induced apoptosis and reduced oxidative stress-induced phosphorylation in JNK and p38 MAPK [105].

In an in vivo model of myocardial ischemia/reperfusion, 20 mg/kg OLE increased the levels of superoxide dismutase (SOD) and reduced glutathione (GSH), while decreasing the lipid peroxidation marker malondialdehyde (MDA). This protective effect was mediated through a mechanism involving the suppression of protein expression of p-IκBα, p53, p-MEK, and p-ERK [64]. In addition, OLE and HT increase CAT and SOD expression and activity in high-fat diet animal models in liver [82] and adipose tissue [106]. Similar results regarding the OLE and HT-mediated increase in tissue expression and activity of catalase (CAT) and superoxide dismutase (SOD), as well as improvements in tissue levels of glutathione (GSH) and reductions in malondialdehyde (MDA) content, were observed in in vivo models of heart failure. These findings suggest a consistent antioxidant effect of both compounds in protecting cardiac tissue [65]. During aging, the main regulator of cellular ROS homeostasis, nuclear factor E2-related factor 2 (Nrf2), and the expression of its target genes generally decrease. This reduction contributes to a decline in the cell’s ability to compensate for oxidative stress, making cells more susceptible to damage from reactive oxygen species (ROS) over time [107]. In vitro, HT promoted Nrf2 nuclear localization in RAW264.7 macrophages [102]. In vivo, OLE increased Nrf2 protein expression [108] while reducing the expression of Nrf2-regulated gene heme oxygenase-1 (HO-1, an antioxidant defense marker) [109,110]. Similarly, in vivo, HT was found to enhance Nrf2 expression and transactivation. Nrf2-dependent gene expression plays a pivotal role in mediating the oxidative stress response in the presence of HT. This includes an increase in the mRNA levels of antioxidant detoxification system components such as glutathione-S-transferase (GST), γ-glutamyl cysteine synthetase (γ-GCS), nicotinamide adenine dinucleotide phosphate (NQO1), sirtuin 1 (SIRT-1), and paraoxonase-2 (PON2), as well as an increase in heme oxygenase-1 (HO-1) protein levels. These changes collectively enhance the cell’s ability to counteract oxidative stress [111,112].

A randomized cross-over study found that the intake of an olive oil phenolic-enriched meal (400 mg/kg) significantly lowered F2-isoprostane levels compared to a meal with low olive oil phenolic content (80 mg/kg). F2-isoprostanes are produced as a result of free radical-induced peroxidation of arachidonic acid, a common membrane-bound fatty acid, and serve as a marker of oxidative stress. This suggests that higher phenolic content in olive oil may contribute to a reduction in oxidative damage [113].

The antioxidant properties of HT are attributed to its ability to scavenge free radicals and stimulate the synthesis of antioxidant enzymes, such as superoxide dismutase and catalase, which also help limit the lipid peroxidation of LDL cholesterol. In a study by Ikonomidis et al., a significant reduction in malondialdehyde (MDA), an oxidative stress biomarker indicating lipid peroxidation, as well as circulating levels of oxidized LDL, was observed following the administration of OOHT. This suggests that HT can effectively mitigate oxidative damage and its associated effects on lipid metabolism [99]. Additionally, oleuropein, a constituent of olive, demonstrated significant anti-ischemic and antioxidant properties. It was also found to reduce circulating lipids in animal models of experimental myocardial infarction. These findings highlight oleuropein’s potential as a protective agent against ischemic damage and its role in modulating lipid profiles in cardiovascular conditions [114]. MDA, produced during fatty acid oxidation, was used as an index of serum lipid peroxide concentration, while serum 3-nitrotyrosine levels were measured as an indicator of peroxynitrite. The results suggest that HT may be less effective in scavenging peroxynitrite compared to hydroxyl radicals (•OH). Consequently, a short-term intervention, such as a 1-month therapy, may not be long enough to achieve the maximum inhibitory effect of HT, particularly regarding peroxynitrite scavenging [115].

Cardiac hypertrophy (CH) is a common condition linked to various factors, including obesity, hypertension, and aging [116,117]. Models of aging induced by D-galactose (D-GAL) in rats, which closely mimic natural aging, exhibit structural changes in the heart that reflect age-related cardiac decline [118]. Olive oil (OO) has been shown to modulate cardiac hypertrophy and dysfunction associated with myocardial infarction through its potent antioxidant and anti-inflammatory properties [119]. Findings by Shahidi et al. revealed that aging rats experience elevated levels of malondialdehyde (MDA), an oxidative stress marker, alongside reduced activity of superoxide dismutase (SOD), an antioxidant enzyme. Treatment with olive leaf extract (OLO) significantly improved MDA and SOD levels, demonstrating its antioxidative effects on cardiac tissue [120]. These protective mechanisms are further attributed to the activation of SIRT1 and PGC1-α pathways, which play a crucial role in cellular resilience and metabolic regulation improving mitochondrial biogenesis [121,122].

## 6. Effect on Endothelial Dysfunction

Endothelial dysfunction, a key mechanism underlying the progression of arteriosclerotic disease, is widely acknowledged as a critical indicator of cardiovascular risk [123]. It is mainly characterized by the decreased bioavailability of nitric oxide (NO) and increased levels of oxidized low-density lipoprotein (ox-LDL) [124]. A major mechanism contributing to nitric oxide (NO) inactivation involves the disruption of the l-arginine–NO pathway by oxidative stress, resulting in increased plasma levels of asymmetric dimethylarginine (ADMA), which further amplifies oxidative stress [125]. Phenolic compounds derived from olive oil (OO) have demonstrated protective effects on the endothelium, as shown in in vitro studies involving typical OO phenolic compounds, while evidence from in vivo circulating metabolites remains more limited. Specifically, OO phenolic extracts, oleuropein aglycone, and homovanillic alcohol (a hydroxytyrosol metabolite) have been shown to inhibit the surface expression of vascular cell adhesion molecule-1 (VCAM-1), intercellular adhesion molecule-1 (ICAM-1), and E-selectin in human umbilical vascular endothelial cells following stimulation with the pro-inflammatory cytokine TNF-α [126]. Moreno et al. observed that the blood pressure (BP)-lowering effects of a polyphenol-rich olive oil diet were more pronounced in individuals with higher baseline BP. This finding is particularly noteworthy given that the study focused on participants with low cardiovascular risk, specifically young women with high-normal BP or stage 1 hypertension. It is plausible that the BP-lowering effects mediated by olive oil polyphenols could be even more significant in patients with more severe hypertension.

Furthermore, after adherence to the polyphenol-rich olive oil diet, reductions in nitrites/nitrates, asymmetric dimethylarginine (ADMA), oxidized low-density lipoprotein (ox-LDL), and C-reactive protein (CRP) were closely correlated with their baseline serum or plasma levels, with the greatest benefits observed in participants presenting the poorest initial values [21]. The maintenance of proper endothelial function depends on a delicate balance between mechanisms that preserve its integrity and those that facilitate repair and recovery from dysfunction, a state referred to as vascular endothelial homeostasis. This concept encompasses the equilibrium in reactive oxygen species (ROS) production, a reduction in the pro-apoptotic environment, and the capacity for endothelial regeneration through angiogenesis. These critical biological processes are modulated by epigenetic factors, such as microRNAs (miRNAs), and are mediated by specific regulatory proteins [127]. The CARDIOPREV study [128], that is an observational study, has demonstrated the efficacy of a Mediterranean diet, rich in MUFAs from olive oil, in improving endothelial dysfunction and addressing various cardiovascular risk factors—including blood lipids, glucose levels, and inflammation—has been well demonstrated. Additionally, its role in reducing cardiovascular events, both in primary and secondary prevention, can be attributed not only to its favorable fatty acid profile, but also to its high antioxidant content. Key phenolic compounds, such as hydroxytyrosol, tyrosol, phenyl alcohols, and flavonoids, are particularly instrumental in these protective effects [9,10,11,12,13,14,15,16,17,18,19,20,21,22,23,24,25,26,27,28,29,30,31,32,33,34,35,36,37,38,39,40,41,42,43,44,45,46,47,48,49,50,51,52,53,54,55,56,57,58,59,60,61,62,63,64,65,66,67,68,69,70,71,72,73,74,75,76,77,78,79,80,81,82,83,84,85,86,87,88,89,90,91,92,93,94,95,96,97,98,99,100,101,102,103,104,105,106,107,108,109,110,111,112,113,114,115,116,117,118,119,120,121,122,123,124,125,126,127,128,129]. An increase in endothelial repair mechanisms, including cellular proliferation and angiogenesis, was observed following adherence to the Mediterranean diet, alongside a reduction in processes associated with endothelial damage, such as in vitro reactive oxygen species (ROS) production, cellular senescence, and apoptosis. Notably, these benefits were evident regardless of the severity of the pre-existing endothelial dysfunction [130,131].

Several mechanisms may explain how olive oil (OO) improves endothelial function, contributing to the beneficial effects observed in this study. One primary mechanism is the enhancement of nitric oxide (NO) bioavailability, which plays a critical role in maintaining vascular health and endothelial function [132], which is essential for healthy endothelial function due to its vasodilatory, antiatherogenic, and antiproliferative actions [133]. The NO ‘boostingș effects of the OO may be due to antioxidant effects minimizing superoxide scavenging of NO [134]. In addition, the OO may improve endothelial function by reducing the oxidation of LDL [135], which plays a major role in endothelial dysfunction and atherogenesis [136,137]. The reduced concentrations of oxidized LDL associated with olive oil (OO) consumption are likely attributable to the antioxidant properties of this dietary pattern, combined with the increased intake of monounsaturated fatty acids (MUFAs), which enhance LDL resistance to oxidation. Furthermore, a well-established connection exists between inflammation and endothelial dysfunction, underscoring the importance of anti-inflammatory interventions in preserving endothelial health [138], and several studies have demonstrated beneficial effects of the OO on inflammatory markers including IL-6, C-reactive protein (CRP), TNF-α, vascular cell adhesion protein-1 (VCAM-1), and soluble intercellular adhesion molecule-1 (sICAM-1) [139,140]. These effects are associated with the downregulation of the NF-kB pathway [141] and altered methylation of inflammation-related genes [142], and may further contribute towards improvements in endothelial function with a MedDiet.

Matrix metalloproteinases (MMPs) are integral to endothelial repair processes. In both human and experimental atherosclerosis, macrophages co-localize with and secrete active MMPs, including gelatinase MMP-9. This enzyme is specialized in degrading basement membrane collagens and elastin, playing a significant role in atherogenesis, unstable coronary syndromes, and the development of aortic aneurysms. Growing evidence highlights MMPs as key molecular mediators in the pathogenesis of arterial diseases [143]. Collagens, types 1 and 3, are the main proteins in arterial walls being also present in the thickened intima of atherosclerotic lesions [144,145]. Fragments of collagen, particularly types I and III, found in urine are indicative of proteolytic activity within the arterial walls and other vascular structures. These collagen fragments are upregulated in the urine of patients with coronary artery disease (CAD), reflecting the ongoing degradation of the extracellular matrix in the context of vascular pathology [146]. Increase in collagen degradation is related with an increase on collagenases circulation, such as MMP-9, as shown in patients with CAD.

In an in vitro study, hydroxytyrosol (1–10 μm) reduced MMP-9 (IC_50_ = 10 μm/L, *p* < 0.05) and COX-2 induction in activated human monocytes with phorbol myristate acetate [147]. These effects were mediated through the inhibition of the transcription factor NF-κB and the activation of protein kinase Cα and protein kinase Cβ1. The findings align with previous in vitro studies, which demonstrated that olive oil (OO) phenolics, such as hydroxytyrosol and oleuropein aglycone, can inhibit MMP-9 activity in endothelial cells. Hydroxytyrosol was shown to inhibit MMP-9 in phorbol myristate acetate-induced cells, while oleuropein aglycone exerted similar effects in TNF-α-induced cells, both acting through the NF-κB pathway. Notably, hydroxytyrosol did not exhibit activity on MMP-9 in TNF-α-induced cells [148,149]. The discriminatory polypetides that increase in CAD include collagen type 1 and 3 fragments with a C-terminal GxPGP motif [150]. An increase in these polypeptides could result from a decrease in protease activity, potentially linked to chemical alterations of the substrate (e.g., oxidative damage), which would inhibit the protease from acting at its specific site. Alternatively, a reduction in circulating levels may occur due to the lack of enzyme activation. Matrix metalloproteinase-2 (MMP-2) is secreted in an inactive form (pro-MMP-2), and several factors, such as plasmin [151] and thrombin [152], can promote its activation, thereby contributing to its biological activity. Other mechanisms that involve proteinases or oxidative stress can also activate MMP-2 [153].

## 7. Antiaggregant Effect

Hydroxytyrosol acetate (HT-AC) is a polyphenolic compound found in virgin olive oil (VOO) in quantities comparable to hydroxytyrosol (HT). Several phenolic compounds in VOO, including HT, oleuropein, and phenolic isochromans, have been shown to inhibit platelet function. These compounds contribute to the potential cardiovascular benefits of VOO by modulating platelet activity, which is crucial in the prevention of thrombotic events [154,155]. In fact, feeding VOO to rats or rabbits was found to significantly reduce collagen-induced platelet aggregation [156,157]. González-Correa et al. demonstrated that hydroxytyrosol acetate (HT-AC) has a stronger antiaggregating effect than hydroxytyrosol (HT) and is nearly as effective as acetylsalicylic acid (ASA) in blocking platelet aggregation. HT-AC was shown to inhibit thromboxane production more than prostacyclin production, which may have significant benefits for preventing thrombosis. The cyclooxygenase-inhibiting mechanism appears to play a key role, as HT has no effect on other platelet pathways, such as cAMP at concentrations of 100 μM [158].

Additionally, HT-AC increased aortic nitric oxide (NO) metabolites and red blood cell glutathione (GSH) levels, suggesting that the antioxidant action and NO release activation are likely mechanisms contributing to its anti-aggregating effects at the endothelial level [159].

## 8. Effect on Blood Pressure

**Figure 2:**
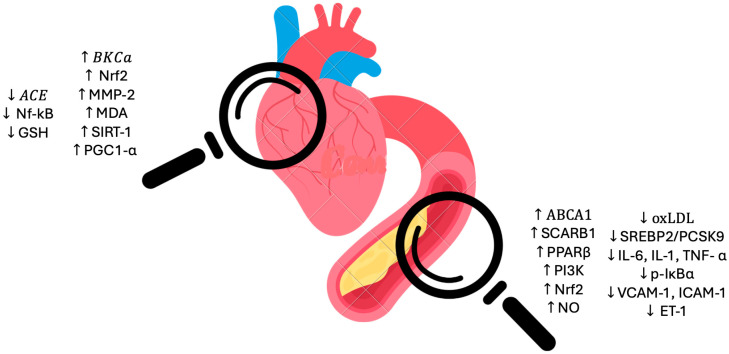
Main molecular pathways involved at the cardiac and vascular level.

The relationship between extra-virgin olive oil (EVOO) and hypertension is another important aspect to consider. In their trial, Martín-Peláez et al. demonstrated that daily consumption of EVOO rich in phenolic compounds led to a reduction in systolic blood pressure (SBP). This effect was attributed to the inhibition of genes associated with the renin–angiotensin–aldosterone system, specifically the angiotensin-converting enzyme (ACE) and nuclear receptor subfamily 1 group H member 2 (NR1H2) genes [160]. Loizzo et al., in their paper, proved that phenolic compounds found in EVOO could inhibit ACE’s action [161] and proposed the ability of flavonoids to engender chelate complexes with zinc ions within the active center of ACE as a possible mechanism [162]. A secondary analysis of the landmark PREDIMED interventional trial revealed that participants adhering to either variation in the Mediterranean diet (MedDiet), enriched with extra-virgin olive oil (EVOO) or nuts, experienced significant reductions in both systolic and diastolic blood pressure (DBP). Additionally, nitric oxide (NO) concentrations, a potent vasodilator, were elevated. These findings suggest an additional antihypertensive mechanism associated with the consumption of EVOO, potentially contributing to its beneficial effects on blood pressure regulation [163]. Similarly, Storniolo et al. corroborated these findings, emphasizing that EVOO’s ability to lower blood pressure is primarily attributed to the upregulation of nitric oxide (NO) synthesis and the downregulation of caveolin-2 expression. This effect was particularly pronounced in hypertensive women, further supporting the antihypertensive potential of EVOO in this population [164]. In addition to enhancing nitric oxide (NO) production, the polyphenols in extra-virgin olive oil (EVOO) seem to play a protective role in maintaining endothelial integrity and mitigating blood pressure. This effect is mediated by the suppression of endothelin-1 (ET-1), a well-established vasoconstrictor peptide, further contributing to the antihypertensive properties of EVOO [165]. In Figure 2, the main molecular pathways involved at the cardiac and vascular level.

Moreover, polyphenol-rich olive oil has been shown to effectively reduce blood pressure while addressing several key factors contributing to endothelial dysfunction, including elevated serum asymmetric dimethylarginine (ADMA), oxidized low-density lipoprotein (ox-LDL), and plasma C-reactive protein (CRP) levels. Additionally, its consumption has been associated with improved hyperemic responses following ischemia, further supporting its beneficial effects on endothelial function and vascular health [21]. Another potential antihypertensive mechanism was proposed by D’Agostino et al., who demonstrated that phenolic compounds in extra-virgin olive oil (EVOO) could induce vasodilation in the mesenteric arteries of rats. This effect was mediated through the activation of large-conductance calcium-activated potassium (BKCa) channels, which were triggered by an increase in intracellular calcium levels. The elevation in calcium was attributed to enhanced calcium influx across the plasma membrane and its release from sarcoplasmic reticulum calcium stores, suggesting a direct mechanism through which EVOO phenolics could exert vasodilatory effects [166]. Additionally, research by Hidalgo et al. suggested that EVOO-induced modifications in the gut microbiota of hypertensive rats, particularly an increase in specific bacterial populations, were linked to a reduction in systolic blood pressure (SBP). This highlights a potential gut microbiota-mediated mechanism through which EVOO may exert its antihypertensive effects [1,2,3,4,5,6,7,8,9,10,11,12,13,14,15,16,17,18,19,20,21,22,23,24,25,26,27,28,29,30,31,32,33,34,35,36,37,38,39,40,41,42,43,44,45,46,47,48,49,50,51,52,53,54,55,56,57,58,59,60,61,62,63,64,65,66,67,68,69,70,71,72,73,74,75,76,77,78,79,80,81,82,83,84,85,86,87,88,89,90,91,92,93,94,95,96,97,98,99,100,101,102,103,104,105,106,107,108,109,110,111,112,113,114,115,116,117,118,119,120,121,122,123,124,125,126,127,128,129,130,131,132,133,134,135,136,137,138,139,140,141,142,143,144,145,146,147,148,149,150,151,152,153,154,155,156,157,158,159,160,161,162,163,164,165,166,167].

The findings indicate that the cardioprotective properties of long-chain fatty acids in this animal model are influenced by both their saturation level and the configuration of cis/trans double bonds, particularly at high doses. Similar positive effects were observed in a hypertension model using spontaneously hypertensive rats, where the intake of virgin olive oil (VOO) and oleic acid led to beneficial outcomes, further supporting the role of olive oil-derived fatty acids in cardiovascular protection [168]. The molecular mechanisms underlying the effects of oleic acid on blood pressure regulation were investigated by analyzing signaling proteins in the aorta. Dietary olive oil (OO) leads to increased incorporation of oleic acid into cellular membranes, thereby altering their lipid structure. This modification modulates G-protein-mediated signaling pathways, which ultimately contribute to blood pressure reduction [169]. Unlike its structural analogs, elaidic and stearic acid, due to its cis-18:1n-9 configuration, oleic acid specifically influences the organization of membrane lipids and regulates the α22-adrenoceptor system involved in blood pressure control. This system operates through the α22A/D-adrenoceptor/G protein/adenylyl cyclase-cAMP/PKA signaling cascade, as demonstrated in both in vitro and in vivo models. This unique mechanism underscores the potential role of oleic acid in modulating vascular function and blood pressure regulation [170]. Oleic acid also supports cardiac health by modulating intramyocardial triacylglycerol (TAG) turnover. In pressure-overloaded failing hearts, where TAG dynamics are typically impaired, oleate (a derivative of oleic acid) was shown to enhance these dynamics compared to palmitate (a derivative of palmitic acid, a major saturated fatty acid in palm oil). This improvement highlights the crucial role of intracellular lipid storage types in regulating nuclear receptor signaling and myocardial contractility, particularly in diseased hearts [171]. Nitric oxide (NO), a key mediator of vascular relaxation, interacts with lipids and proteins to form bioactive compounds, such as nitro-fatty acids. These molecules exert significant cardiovascular effects by promoting vasodilation, reducing platelet activation, and mitigating inflammation, thereby contributing to overall cardiovascular health and function [172,173].

Both oleic acid and linoleic acid, when reacting with nitrite, can generate nitro-fatty acids. In a mouse model, nitro-oleic acid demonstrated antihypertensive effects by inhibiting soluble epoxide hydrolase, which led to a reduction in blood pressure in angiotensin II-induced hypertension. This highlights the potential therapeutic role of nitro-fatty acids in managing hypertension [174]. In the context of cardiometabolic diseases, it is important to recognize that, similar to hypertension (HT), the consumption of certain compounds may also lead to adverse effects, especially at higher doses. For example, research by Sun and colleagues demonstrated that oleuropein (OLE), administered at 60 mg/kg/day over eight weeks, effectively reduced blood pressure by targeting the hypothalamus and modulating pathways involving Nrf-2 and associated genes. This effect was observed in spontaneously hypertensive rats, underscoring both the therapeutic potential of oleuropein and the critical importance of dose management to ensure safety [175].

## 9. Conclusions

**Figure 3:**
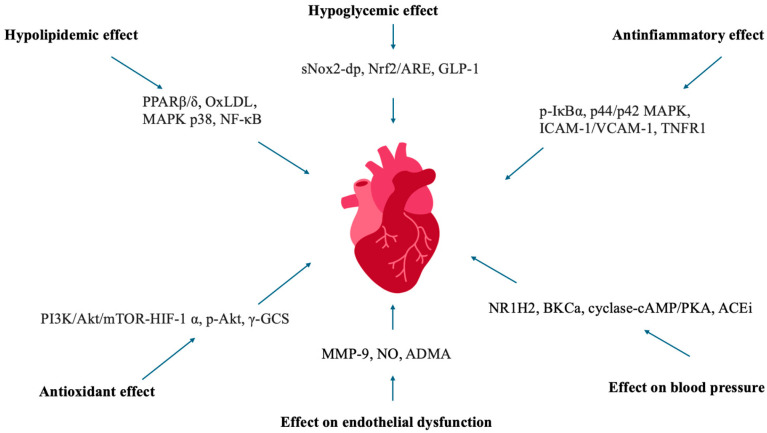
Main pathways involved in cardiovascular health.

The protective effects of olive oil on cardiovascular health are well-documented in both epidemiological and experimental studies. Its high content of monounsaturated fatty acids (MUFAs), particularly oleic acid, alongside bioactive compounds such as polyphenols, contributes to its cardioprotective properties. These components have demonstrated anti-inflammatory, antioxidant, and lipid-lowering effects, which collectively reduce the risk of atherosclerosis and coronary heart disease. Moreover, olive oil intake is associated with improved endothelial function and reduced blood pressure, further enhancing its cardiovascular benefits. Intervention trials and meta-analyses consistently highlight the role of olive oil, particularly extra-virgin olive oil (EVOO), as a cornerstone of the Mediterranean diet, which is linked to a lower incidence of cardiovascular events. However, while the evidence is robust, it is crucial to consider individual variability in response to dietary interventions, as genetic and lifestyle factors may influence outcomes. Future research should focus on elucidating the molecular mechanisms underlying these effects and exploring personalized nutritional approaches. In conclusion, incorporating olive oil into a balanced diet represents a simple, yet effective, strategy for promoting cardiovascular health, reinforcing the importance of dietary choices in preventing chronic diseases and improving overall quality of life. In Figure 3, the main pathways involved in cardiovascular health are shown.

Although numerous studies have explored the beneficial effects of olive oil on cardiovascular health, several limitations must be acknowledged. Many investigations rely on observational designs, which inherently restrict the ability to draw definitive causal inferences. Additionally, the heterogeneity of study populations and variations in olive oil composition, particularly in terms of polyphenol content and differences in dietary patterns, make it challenging to isolate the specific impact of olive oil from the overall Mediterranean diet.

Another significant limitation is the inconsistency in methodologies used to measure cardiovascular outcomes such as LDL oxidation, inflammatory markers, and endothelial function. These variations can lead to discrepancies in the reported effects and hinder the comparison of results across different studies. Moreover, the bioavailability of olive oil’s bioactive compounds may differ based on factors such as geographic origin, processing methods, and storage conditions, further complicating the interpretation of study findings.

Despite these limitations, evidence suggests that the beneficial impact of olive oil on cardiovascular risk is most pronounced in specific high-risk populations. In particular, individuals who are obese, diabetic, or suffering from chronic coronary syndrome seem to derive greater cardiovascular protection from olive oil consumption. In these patients, the potent antioxidant and anti-inflammatory properties of olive oil’s polyphenols may contribute more significantly to reducing cardiovascular risk by improving endothelial function and attenuating lipid oxidation.

Future research should aim to overcome these limitations through well-designed, randomized controlled trials with standardized methodologies and more homogeneous populations. Such studies will be crucial for clarifying the mechanisms underlying olive oil’s cardioprotective effects and for establishing more definitive dietary recommendations for high-risk patient groups.

## Figures and Tables

**Table 1:** Olive oil components and their effect on cardiovascular health.

Olive Oil Component	Cardiovascular Effects/Mechanisms
**Monounsaturated fatty acids (MUFAs), predominantly oleic acid**	Improve lipid profiles by reducing LDL oxidation and enhancing endothelial function, thereby lowering cardiovascular risk.
**Phytosterols**	Reduce cholesterol absorption and improve serum lipid profiles, contributing to a decreased risk of atherosclerosis.
**Triterpenes, squalene, and pigments (unsaponifiable fraction)**	Exhibit antioxidant properties that help protect against oxidative stress and endothelial dysfunction.
**Polyphenols (secoiridoids)** *(e.g.,* *o* *leuropein aglycone, oleacein, deacetoxyoleuropein, oleocanthal)*	Possess potent antioxidant and anti-inflammatory activities that reduce LDL oxidation and support vascular health.
**Phenolic alcohols** *(hydroxytyrosol, tyrosol)*	Act as powerful antioxidants, mitigating oxidative stress and lipid peroxidation, which plays a key role in lowering cardiovascular risk.
**Phenolic acids** *(gallic acid, protocatechic acid, p-hydroxybenzoic acid, vanillic acid, caffeic acid, syringic acid, p- and o-coumaric acid, ferulic acid, cinnamic acid)*	Contribute to the overall antioxidant capacity, protecting against the oxidative modification of lipids and vascular inflammation.
**Flavonoids** *(*e.g.*, luteolin, apigenin)*	Provide anti-inflammatory and antioxidant effects, thereby improving vascular function and reducing the progression of atherosclerosis.
**Lignans** *(acetoxypinoresinol, pinoresinol)*	Offer additional antioxidant and anti-inflammatory benefits, further supporting cardiovascular health.

## Data Availability

No new data were created or analyzed in this study.

## Abbreviations

The following abbreviations are used in this manuscript:

MedDiet	Mediterranean diet
MD	Mediterranean diet
MUFA	Monounsaturated fatty acid
ROO	Refined olive oil
EVOO	Extra-virgin olive oil
CVD	Cardiovascular disease
OO	Olive oil
OLC	Oleocanthal
OLE	Oleuropein aglycone
HT	Hydroxytyrosol
TYR	Tyrosol
EFSA	European Food Safety Authority
SCSCD	Seven Countries Study of Cardiovascular Disease
CHD	Coronary heart disease
PPAR	Peroxisome proliferator-activated receptors
OOPC	Olive oil polyphenol concentrate
HDL	High-density lipoprotein
LDL	Light density lipoprotein
OxLDL	Oxidized light-density lipoprotein
LOX-1	Lectin-like oxidized receptor 1
ROS	Reactive oxygen species
SREBP	Sterol regulatory element-binding protein 2
PCSK9	Proprotein convertase subtilisin/kexin type 9
T2D	Type 2 diabetes
HbA1c	Glycated hemoglobin A1c
JNK	Jun N-terminal kinase
AGEs	Advanced glycosylated end-products
sNox2-dp	Soluble NADPH oxidase-derived peptide
HOMA-IR	Homeostatic model assessment of insulin resistance
TACE	Tumor necrosis factor converting enzyme
SOD	Superoxide dismutase
COX	Cyclooxygenase
CAT	Catalase
MDA	Malondialdehyde
D-GAL	D-galactose
CH	Cardiac hypertrophy
VCAM-1	Vascular cell adhesion molecule-1
ICAM-1	Intracellular adhesion molecule-1
ADMA	Asymmetric dimethylarginine
NO	Nitric oxide
MMP	Matrix metalloproteinas
CAD	Coronary artery disease
HT-AC	Hydroxytirosol acetate
BKCa	Calcium-activated potassium channels
